# Association Between Smoking, Smoking Cessation, and Mortality by Race, Ethnicity, and Sex Among US Adults

**DOI:** 10.1001/jamanetworkopen.2022.31480

**Published:** 2022-10-24

**Authors:** Blake Thomson, Jonathan Emberson, Ben Lacey, Sarah Lewington, Richard Peto, Ahmedin Jemal, Farhad Islami

**Affiliations:** 1 Department of Surveillance and Health Equity Science, American Cancer Society, Atlanta, Georgia; 2 Nuffield Department of Population Health, University of Oxford, Oxford, United Kingdom; 3 Medical Research Council Population Health Research Unit, Nuffield Department of Population Health, University of Oxford, Oxford, United Kingdom; 4 UKM Medical Molecular Biology Institute, Universiti Kebangsaan Malaysia, Kuala Lumpur, Malaysia

## Abstract

**Importance:**

Patterns of cigarette smoking and smoking cessation vary considerably across demographic groups in the US, but there is limited evidence on whether the hazards of smoking and benefits of quitting vary across these groups. Population-specific evidence on the benefits of quitting smoking may motivate cessation among groups historically underrepresented in medical research.

**Objective:**

To quantify the association between smoking, smoking cessation, and mortality by race, ethnicity, and sex.

**Design, Setting, and Participants:**

This nationally representative, prospective cohort study used data from the US National Health Interview Survey collected via questionnaire between January 1997 and December 2018 among adults aged 25 to 84 years at recruitment. Participants were followed up for cause-specific mortality through December 31, 2019.

**Exposures:**

Self-reported smoking status at recruitment, age at quitting smoking, and years since quitting smoking.

**Main Outcomes and Measures:**

The main outcomes were all-cause mortality and mortality from cancer, cardiovascular disease, and lower respiratory disease. Adjusted mortality rate ratios comparing never, former, and current smokers were calculated using Cox proportional hazards regression. Weighted analyses were conducted by race, ethnicity, and sex as reported by participants.

**Results:**

Among the 551 388 participants in the main analyses, the mean (SD) age at recruitment was 48.9 (15.3) years; 307 601 (55.8%) were women, 87 207 (15.8%) were Hispanic, 75 545 (13.7%) were non-Hispanic Black, 355 782 (64.5%) were non-Hispanic White, and 32 854 (6.0%) identified as other non-Hispanic race and ethnicity. There were 74 870 deaths among participants aged 25 to 89 years during follow-up (36 792 [49.1%] among men; 38 078 [50.9%] among women). The all-cause mortality rate ratio (RR) for current vs never smoking was 2.80 (95% CI, 2.73-2.88) overall. The RRs were similar by sex but varied by race and ethnicity: Hispanic, 2.01 (95% CI, 1.84-2.18); non-Hispanic Black, 2.19 (95% CI, 2.06-2.33); non-Hispanic White, 3.00 (95% CI, 2.91-3.10); and other non-Hispanic race and ethnicity, 2.16 (95% CI, 1.88-2.47). When comparing those who quit smoking before age 45 years with never smokers, all-cause mortality RRs were 1.15 (95% CI, 1.03-1.28) among Hispanic individuals, 1.16 (95% CI, 1.07-1.25) among non-Hispanic Black individuals, 1.11 (95% CI, 1.08-1.15) among non-Hispanic White individuals, and 1.17 (95% CI, 0.99-1.39) among other non-Hispanic individuals.

**Conclusions and Relevance:**

In this prospective cohort study, among men and women from diverse racial and ethnic groups, current smoking was associated with at least twice the all-cause mortality rate of never smoking. Quitting smoking, particularly at younger ages, was associated with substantial reductions in the relative excess mortality associated with continued smoking.

## Key Points

**Question:**

What are the contemporary hazards of cigarette smoking and benefits of quitting smoking by race, ethnicity, and sex among US adults?

**Findings:**

In this prospective cohort study of 551 388 US adults, smoking was associated with appreciably greater all-cause mortality compared with never smoking irrespective of race, ethnicity, or sex. Quitting smoking was associated with a reduction in the excess mortality associated with continued smoking, with larger reductions among those who quit at younger ages.

**Meaning:**

In this study, among men and women from diverse racial and ethnic groups, quitting smoking was associated with large reductions in the excess mortality associated with continued smoking.

## Teaser

This cohort study assesses associations of cigarette smoking and quitting smoking with mortality by race, ethnicity, and sex among US adults aged 25 to 84 years.

## Introduction

Cigarette smoking continues to cause nearly 530 000 deaths^1^ in the US each year, predominantly from neoplastic, cardiovascular, and respiratory causes. Despite substantial evidence of the hazards of smoking and benefits of quitting,^2,3,4,5,6,7^ there are still 34 million adults who smoke cigarettes^8^ in the US.

Although smoking is well established as a risk factor for mortality in the US population overall, there is limited evidence of its association with mortality in different demographic groups, in which patterns of smoking and smoking cessation vary markedly.^9,10,11^ Quantifying the race-, ethnicity-, and sex-specific associations of smoking with mortality is critical both for health policy planning and for providing targeted public health messages (particularly the benefits of quitting smoking), especially for groups historically underrepresented in medical research.

Whether the hazards of smoking and benefits of quitting are similar among men and women from diverse racial and ethnic groups in the contemporary US remains unclear. Therefore, the aim of this study was to quantify the association, by race, ethnicity, and sex, of cigarette smoking and smoking cessation with all-cause and cause-specific mortality in a nationally representative, contemporary US population.

## Methods

### Study Design and Participants

In this prospective cohort study, we used publicly available data from the US National Health Interview Survey^12^ collected between January 1997 and December 2018 and linked to the National Death Index for mortality follow-up.^13^ The National Health Interview Survey is a questionnaire-based health survey conducted among the civilian, noninstitutionalized US population annually. The survey is conducted using a multistage, complex probabilistic design to ensure that it is nationally representative overall and for demographic subpopulations, including by age, sex, race, and ethnicity. Participants were followed up for mortality through December 31, 2019, by probabilistic linkage to the National Death Index, with more than 95% of adult participants providing sufficient information for mortality follow-up. The main outcomes were all-cause mortality and deaths from 3 major causes (classified by the *International Statistical Classification of Diseases and Related Health Problems, Tenth Revision*): cancer (C00-C97), cardiovascular disease (I00-I09, I11, I13, and I20-I51 [cardiac] and I60-I69 [cerebrovascular]), and lower respiratory disease (J40-J47; hereafter, ‘respiratory’). Because the data were publicly available and deidentified, this study was considered non–human participants research; it was exempt from institutional review board approval and informed consent was waived in accordance with the US federal regulation (45 CFR §46). This study followed the Strengthening the Reporting of Observational Studies in Epidemiology (STROBE) reporting guideline.

### Exposure Categories

Analyses were conducted to quantify the overall and sex-, race-, and ethnicity-specific hazards of cigarette smoking and benefits of quitting in the US. Participants were considered never smokers if they had not smoked at least 100 cigarettes in their lifetime, and those who had smoked at least 100 cigarettes in their lifetime were classified as ever smokers. To reduce the potential for reverse causality^14^ in prospective analyses, as in previous research,^2^ ever smokers who had reported quitting in the 5 years preceding death were considered current smokers, in addition to those reporting smoking every day or some days at recruitment. Other ever smokers who had quit smoking by recruitment were considered former smokers. The smoking-related questions asked during data collection are given in eTable 1 in the Supplement.

Overall and sex-specific analyses of former smokers were conducted to provide information about the benefits of quitting smoking at different ages (<35, 35-44, 45-54, or 55-64 years) and for different durations (5-14, 15-24, 25-34, or ≥35 years). To produce statistically reliable estimates, race- and ethnicity-specific analyses of former smokers were restricted to quitting before age 45 years or at ages 45 to 64 years and quitting for 5 to 14 years or for more than 15 years, and analyses specific to race, ethnicity, and sex were limited to never, former, and current smokers.

### Statistical Analysis

We estimated mortality rate ratios (RRs) for deaths between ages 25 and 89 years among current and former cigarette smokers vs never smokers using Cox proportional hazards regression. Analyses were adjusted for age at risk (in 5-year age groups), educational level completed (less than high school, high school, some college, or college degree), and alcohol consumption (never, former, or current [less than weekly, 1-2 days per week, or ≥3 days per week]). Analyses were further adjusted for self-reported race and ethnicity (Hispanic, non-Hispanic Black [hereafter referred to as Black], non-Hispanic White [hereafter referred to as White], or other non-Hispanic [any group other than Black, Hispanic, or White]) and sex (female or male) where relevant. Categories of race and ethnicity were constructed to align with the US Office of Management and Budget standards^15^ on race and ethnicity reporting, combining categories with too few participants to be statistically reliable.

To estimate the potential relationship of cigarette smoking with mortality in the US population by race, ethnicity, and sex from 1997 to 2018, all-cause and cause-specific population-attributable fractions were also estimated. These estimates were derived from race- and ethnicity-specific ever vs never smoking RRs (adjusted for sex) and the race-, ethnicity-, and sex-specific prevalence (*P*) of individuals ever smoking among those who died of the relevant cause using the following formula: *P* × (RR – 1) divided by RR. The fraction of deaths among ever smokers (ie, attributable fraction) was calculated by setting *P* equal to 1. The percentage of increased mortality risk avoided by quitting (compared with continuing to smoke) was estimated through the equation (RR^a^ − RR^b^)/(RR^a^ − 1), where RR^a^ is the RR for current vs never smoking and RR^b^ is the RR for former vs never smoking, multiplied by 100. Analyses accounted for the complex design of the National Health Interview Survey using strata, primary sampling units, and sample adult weights and were conducted between April 2021 and July 2022 using Stata, version 15.1 (StataCorp LLC) and R, version 4.0.5 (R Project for Statistical Computing). *P* values were 2-sided and considered statistically significant at .05.

## Results

### Participant Characteristics

Among 671 696 adults participating in the National Health Interview Survey from 1997 to 2018, 587 903 (87.5%) were aged 25 to 84 years at recruitment. Of these, 22 811 (3.9%) provided insufficient information for mortality follow-up and 13 704 (2.3%) were excluded because of missing exposure or covariate data. Among the 551 388 adults included in the main analyses, 243 787 (44.2%) were men and 307 601 (55.8%) were women; 75 545 (13.7%) were Black (29 441 men [39.0%] and 46 104 women [61.0%]), 87 207 (15.8%) were Hispanic (38 009 men [43.6%] and 49 198 women [56.4%]), 355 782 (64.5%) were White (161 305 men [45.3%] and 194 477 women [54.7%]), and 32 854 (6.0%) identified as other non-Hispanic race and ethnicity (15 032 men [45.8%] and 17 822 women [54.2%]). The mean (SD) age at recruitment was 48.9 (15.3) years overall, ranging from 43.3 (13.6) years among Hispanic men to 50.9 (15.6) years among White women. Weekly alcohol consumption was more common among men than among women within each racial and ethnic group, ranging from 11.2% among Hispanic women to 44.3% among White men (Table 1).

**Table 1.** Baseline Characteristics of the National Health Interview Survey Cohort Participants From 1997 to 2018

Characteristic	Participants^a^								
	Hispanic		Non-Hispanic Black		Non-Hispanic White		Other non-Hispanic^b^		Total
	Men	Women	Men	Women	Men	Women	Men	Women	
Participants, No.	38 009	49 198	29 441	46 104	161 305	194 477	15 032	17 822	551 388
Deaths	3670 (9.7)	3596 (7.3)	5285 (18.0)	6212 (13.5)	26 566 (16.5)	27 010 (13.9)	1271 (8.5)	1260 (7.1)	74 870 (13.6)
Smoking status									
Never	21 712 (59.7)	37 868 (78.8)	14 977 (55.0)	30 505 (68.9)	72 298 (46.3)	106 805 (56.0)	8364 (56.9)	13 400 (78.7)	305 929 (55.9)
Former	8460 (21.9)	5660 (11.5)	6346 (19.3)	6591 (13.2)	51 722 (31.4)	48 176 (24.3)	3473 (22.9)	2155 (10.4)	132 583 (24.3)
Current	7837 (18.4)	5670 (9.7)	8118 (25.7)	9008 (17.9)	37 285 (22.3)	39 496 (19.7)	3195 (20.2)	2267 (11.0)	112 876 (19.8)
Age, mean (SD), y	43.3 (13.6)	44.7 (14.3)	46.5 (14.3)	47.3 (14.8)	49.9 (15.1)	50.9 (15.6)	45.8 (14.3)	46.3 (14.7)	48.9 (15.3)
Educational level attained									
<High school	15 998 (38.9)	20 499 (38.2)	6232 (17.8)	9798 (18.5)	17 161 (10.2)	20 299 (9.6)	1501 (9.8)	2333 (12.3)	93 821 (14.5)
High school	9395 (25.7)	11 599 (24.1)	9386 (32.6)	13 040 (28.3)	44 254 (27.5)	54 677 (28.2)	2782 (18.4)	3586 (19.8)	148 719 (27.2)
Some college	7726 (21.4)	10 916 (23.0)	8455 (29.6)	14 811 (32.9)	45 771 (27.9)	59 838 (30.5)	3481 (22.8)	4320 (22.9)	155 318 (28.2)
College degree	4890 (14.0)	6184 (14.7)	5368 (20.0)	8455 (20.3)	54 119 (34.4)	59 663 (31.7)	7268 (49.0)	7583 (45.1)	153 530 (30.0)
Alcohol consumption									
Never	6299 (16.2)	21 118 (41.1)	5490 (19.5)	16 317 (34.7)	15 883 (10.0)	37 591 (18.8)	3382 (22.8)	7659 (45.0)	113 739 (19.0)
Former	5736 (14.2)	6400 (13.0)	5959 (18.0)	8385 (17.5)	27 725 (16.4)	32 097 (15.7)	2118 (13.6)	2317 (11.7)	90 737 (15.7)
Current									
<Weekly	13 037 (36.4)	16 396 (34.7)	8184 (29.4)	14 703 (33.0)	45 624 (29.4)	71 603 (37.7)	5015 (34.8)	5292 (29.5)	179 854 (33.6)
1-2 d/wk	9121 (23.8)	4017 (8.5)	5905 (20.5)	4748 (10.5)	38 111 (23.8)	32 109 (17.0)	2780 (18.1)	1745 (9.5)	98 536 (18.7)
≥3 d/wk	3816 (9.5)	1267 (2.7)	3903 (12.6)	1951 (4.3)	33 962 (20.5)	21 077 (10.9)	1737 (10.8)	809 (4.4)	68 522 (13.0)
Age at smoking initiation, y^c^									
<18	3959 (52.8)	2562 (46.4)	3709 (44.3)	3518 (38.7)	21 337 (58.6)	20 423 (53.7)	1329 (42.1)	1076 (48.4)	57 913 (53.5)
18-24	2804 (37.1)	1943 (35.8)	3176 (42.4)	3809 (44.2)	12 846 (35.2)	14 294 (36.7)	1405 (45.4)	833 (37.8)	41 110 (37.2)
≥25	805 (10.1)	992 (17.8)	1037 (13.3)	1507 (17.1)	2389 (6.1)	4105 (9.5)	382 (12.5)	311 (13.8)	11 528 (9.3)
Mean (SD), y	18.0 (5.5)	19.5 (7.1)	19.0 (5.6)	19.8 (6.0)	17.2 (4.7)	18.1 (5.5)	18.8 (5.5)	18.9 (6.4)	18.0 (5.4)
Cigarettes per day, No.^d^									
<10	1931 (40.8)	1881 (51.2)	1704 (29.3)	2568 (38.6)	2922 (9.2)	4986 (15.1)	720 (30.8)	535 (32.1)	17 247 (17.6)
10-19	1569 (33.2)	1171 (31.0)	2426 (39.9)	2711 (40.3)	8807 (29.1)	12 558 (39.0)	861 (36.4)	681 (37.4)	30 784 (34.6)
≥20	1363 (26.0)	738 (17.8)	1986 (30.8)	1492 (21.1)	19 253 (61.7)	15 219 (45.9)	830 (32.8)	506 (30.5)	41 387 (47.8)
Mean (SD), No.	12.0 (10.5)	10.3 (9.5)	13.5 (10.0)	11.8 (10.4)	20.2 (11.6)	16.5 (10.2)	14.3 (12.3)	13.3 (9.6)	17.1 (11.2)
Nondaily smoking among current smokers	2954 (39.7)	1868 (33.3)	1975 (25.5)	2179 (23.7)	6174 (16.4)	6600 (16.3)	765 (22.9)	537 (23.7)	23 052 (19.6)
Age at quitting smoking, mean (SD), y^e^	35.3 (12.3)	36.5 (12.8)	40.4 (13.3)	41.2 (13.3)	38.2 (13.5)	38.0 (14.1)	37.4 (12.5)	36.1 (13.2)	38.0 (13.6)

^a^ Data are presented as the number (percentage) of participants unless otherwise indicated. Unweighted sample size is given for raw numbers, and percentages were weighted using National Health Interview Survey sample weights. Totals may not sum owing to rounding. Characteristics are reported for those aged 25 to 84 years at recruitment and, for those who died, the number of deaths at ages 25 to 89 years.

^b^ Other non-Hispanic race and ethnicity includes any group other than Hispanic, non-Hispanic Black, or non-Hispanic White.

^c^ Of the 112 876 current smokers included in the main analyses, 2325 (2.1%) did not provide information about age at initiation.

^d^ Among participants reporting daily smoking; 406 of the 89 824 daily smokers at recruitment (0.5%) did not report the number of cigarettes smoked per day.

^e^ Among former smokers.

Current cigarette smoking prevalence ranged from 9.7% among Hispanic women to 25.7% among Black men (Table 1). Among individuals who reported current smoking, nondaily smoking was most common among Hispanic individuals (men, 39.7%; women, 33.3%) and was least common among White individuals (men, 16.4%; women, 16.3%). Within each racial and ethnic group, men began smoking at a younger age and reported smoking more cigarettes per day than did women. Among daily smokers, mean (SD) daily cigarette consumption was lowest among Hispanic individuals (men, 12.0 [10.5] per day; women, 10.3 [9.5] per day) and greatest among White individuals (men, 20.2 [11.6] per day; women, 16.5 [10.2] per day). Among former smokers, the mean (SD) age at which participants quit was 38.0 (13.6) years; the mean (SD) age was somewhat higher among Black men (40.4 [13.3] years) and Black women (41.2 [13.3] years).

### Overall and Sex-Specific Associations

There were 74 870 deaths among participants aged 25 to 89 years during 6.0 million person-years in the follow-up period (mean [SD] follow-up time per person, 11 [6] years). Of these, 38 078 deaths (50.9%) occurred among women and 36 792 (49.1%) occurred among men. Most deaths occurred among White participants (53 576 [71.6%]), followed by Black (11 497 [15.4%]), Hispanic (7266 [9.7%]), and other non-Hispanic (2531 [3.4%]) participants.

**Figure 1.**
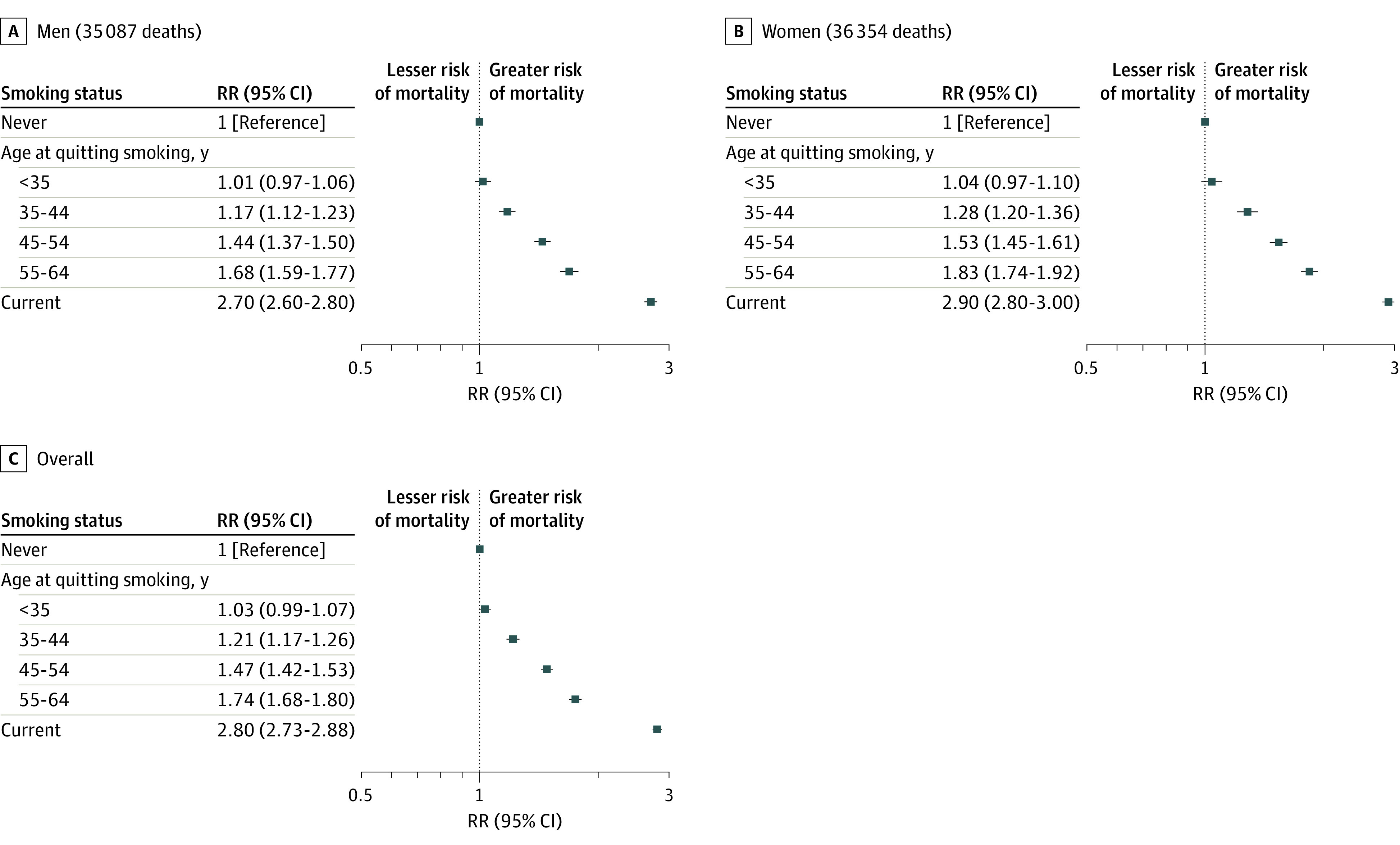
All-Cause Mortality Rate Ratios (RRs) by Age at Quitting Smoking and by Sex Compared With Never and Current Smoking Among Individuals at Risk Aged 25 to 89 Years Adjusted for age at risk, sex, educational level, alcohol consumption, and race and ethnicity. Markers represent RRs, and horizontal lines represent 95% CIs. Lower risk indicates lower risk of mortality, and higher risk indicates higher risk of mortality.

Figure 1 shows the all-cause mortality RRs for current and former cigarette smokers vs never smokers, with a focus on the overall and sex-specific RRs by age at quitting smoking and years since quitting smoking. The all-cause mortality RR for current vs never smoking was 2.80 (95% CI, 2.73-2.88) overall (Figure 1). However, individuals who quit smoking had substantially lower mortality rates than those who continued smoking. Compared with never smokers, the RR for quitting smoking before age 35 years (mean [SD], 26 [4.8] years) was 1.03 (95% CI, 0.99-1.07); at ages 35 to 44 years (mean [SD], 39 [2.9] years), 1.21 (95% CI, 1.17-1.26); at ages 45 to 54 years (mean [SD], 49 [2.8] years), 1.47 (95% CI, 1.42-1.53); and at ages 55 to 64 years (mean [SD], 59 [2.8] years), 1.74 (95% CI, 1.68-1.80). Similarly, compared with never smokers, the RR for individuals who quit smoking 5 to 14 years (mean [SD], 9 [2.7] years) before recruitment was 1.72 (95% CI, 1.65-1.79); 15 to 24 years (mean [SD], 19 [2.7] years), 1.36 (95% CI, 1.31-1.41); 25 to 34 years (mean [SD] 28 [2.7] years), 1.18 (95% CI, 1.14-1.23); and 35 years or more (mean [SD], 42 [6.3] years), 1.04 (95% CI, 1.00-1.08) (eFigure 1 in the Supplement).

The relative excess mortality associated with current smoking was generally similar among men (RR, 2.70; 95% CI, 2.60-2.80) and women (RR, 2.90; 95% CI, 2.80-3.00). These patterns were also observed in analyses of the benefits of smoking cessation at different ages and for different durations.

### Race- and Ethnicity-Specific Associations

**Figure 2.**
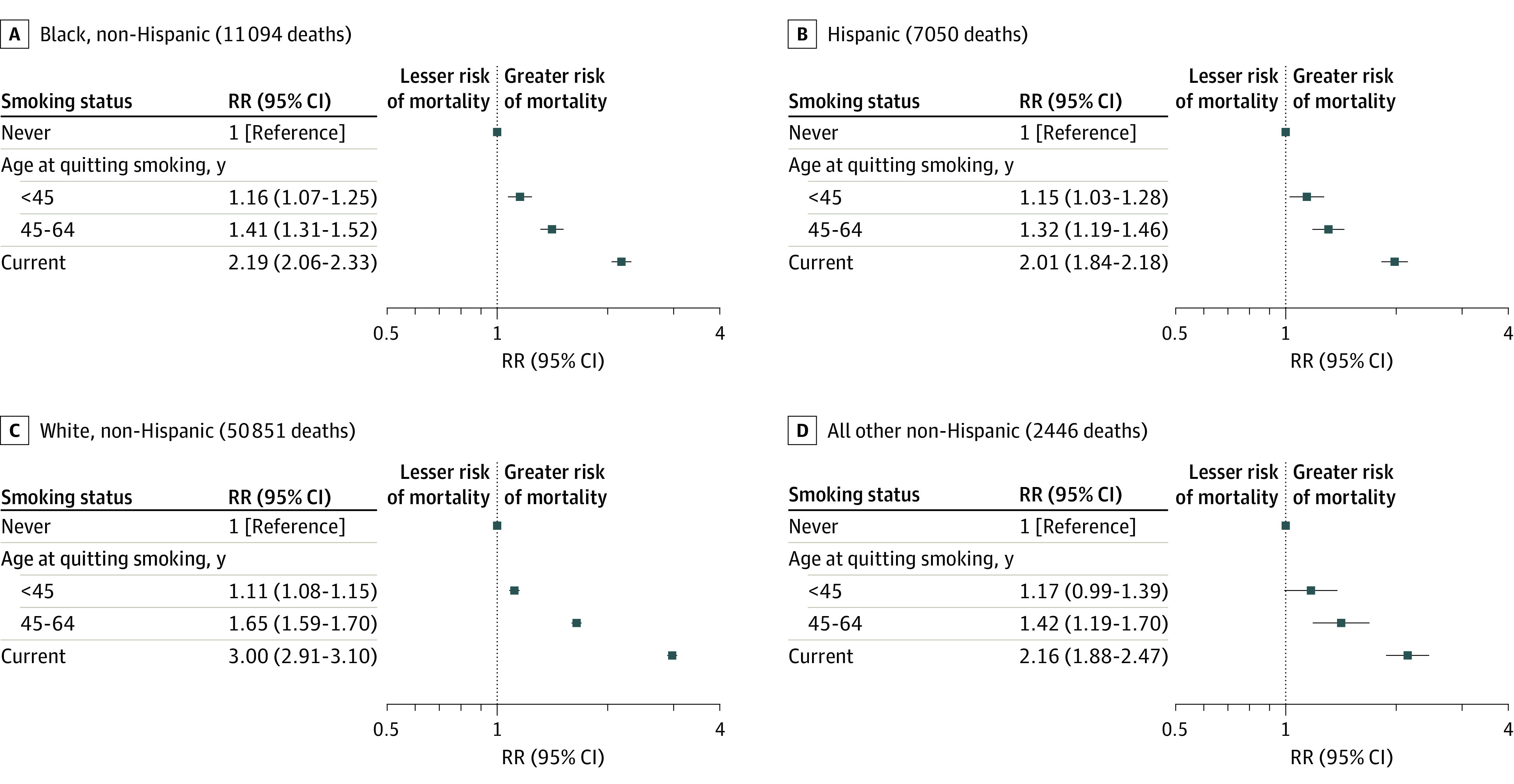
All-Cause Mortality Rate Ratios (RRs) by Age at Quitting Smoking and by Race and Ethnicity Compared With Never and Current Smoking Among Individuals at Risk Aged 25 to 89 Years Adjusted for age at risk, sex, educational level, and alcohol consumption. Other non-Hispanic race and ethnicity includes any group other than Hispanic, non-Hispanic Black, or non-Hispanic White. Markers represent RRs, and horizontal lines represent 95% CIs. Lower risk indicates lower risk of mortality, and higher risk indicates higher risk of mortality.

Although the all-cause mortality RRs among current vs never cigarette smokers varied by race and ethnicity, current smoking was associated with substantial excess mortality in all groups: Black, 2.19 (95% CI, 2.06-2.33); Hispanic, 2.01 (95% CI, 1.84-2.18); White, 3.00 (95% CI, 2.91-3.10); and other non-Hispanic race and ethnicity, 2.16 (95% CI, 1.88-2.47) (Figure 2). However, smoking cessation was associated with substantially lower mortality rates irrespective of race and ethnicity. Compared with never smokers, the all-cause mortality RRs among those who quit smoking before age 45 years (mean [SD], 31 [7.4] years) were 1.16 (95% CI, 1.07-1.25) among Black individuals, 1.15 (95% CI, 1.03-1.28) among Hispanic individuals, 1.11 (95% CI, 1.08-1.15) among White individuals, and 1.17 (95% CI, 0.99-1.39) among individuals who identified as other non-Hispanic race and ethnicity. Thus, quitting smoking before age 45 years was associated with reductions of approximately 90% of the excess risk associated with continued smoking, and quitting at ages 45 to 64 years (mean [SD], 52.8 [5.5] years) was associated with reductions of approximately 66% of this excess risk (Figure 2). The RRs by years since quitting smoking provided similar results: quitting smoking 5 to 14 years (mean [SD], 9.0 [2.7] years) before recruitment was associated with reductions of approximately 50% of the excess mortality among current smokers, and quitting at 15 or more years (mean [SD], 27.7 [10.1] years) before recruitment was associated with reductions of approximately 90% of this excess risk (eFigure 2 in the Supplement).

### Associations by Race, Ethnicity, and Sex 

Table 2 shows the all-cause and cause-specific mortality RRs for current and former vs never cigarette smokers by race, ethnicity, and sex. Although associations varied by race and ethnicity, sex-specific associations were generally similar within racial and ethnic groups. Across all sex-, race-, and ethnicity-specific strata, mortality RRs among former smokers were substantially lower than those among current smokers. Former smokers had approximately 20% the excess all-cause mortality risk of current smokers on average and for each group considered (although statistical power was limited among participants in the other non-Hispanic race and ethnicity group) (Table 2). Thus, among former smokers in each racial and ethnic group, whether male or female, quitting was associated with reductions of approximately 80% of the excess mortality associated with continued smoking (Table 2). These associations were generally consistent for deaths from cancer, cardiovascular disease, and lower respiratory disease. There were insufficient events to examine race-, ethnicity-, and sex-specific associations by age at quitting smoking or time since quitting.

**Table 2.** Adjusted All-Cause and Cause-Specific Mortality RRs for Current or Former Smoking vs Never Smoking by Race, Ethnicity, and Sex^**a**^

Race and ethnicity, smoking status	All-cause		Cancer		Cardiovascular disease		Lower respiratory disease	
	Deaths, No.	RR (95% CI)	Deaths, No.	RR (95% CI)	Deaths, No.	RR (95% CI)	Deaths, No.	RR (95% CI)
**Hispanic men**								
Never	1409	1 [Reference]	303	1 [Reference]	424	1 [Reference]	25	1 [Reference]
Former	1244	1.21 (1.08-1.35)	278	1.21 (0.97-1.51)	422	1.28 (1.06-1.54)	47	2.18 (1.10-4.34)
Current	1017	1.99 (1.77-2.24)	271	2.17 (1.74-2.72)	262	1.96 (1.58-2.43)	44	6.87 (3.64-12.97)
**Hispanic women**								
Never	2407	1 [Reference]	600	1 [Reference]	679	1 [Reference]	55	1 [Reference]
Former	622	1.31 (1.16-1.48)	152	1.12 (0.88-1.41)	163	1.21 (0.97-1.52)	29	3.80 (1.86-7.77)
Current	567	2.05 (1.80-2.33)	157	2.14 (1.68-2.72)	145	2.08 (1.63-2.64)	31	6.57 (3.84-11.23)
**Non-Hispanic Black men**								
Never	1605	1 [Reference]	328	1 [Reference]	541	1 [Reference]	33	1 [Reference]
Former	1672	1.31 (1.21-1.43)	431	1.46 (1.20-1.76)	586	1.25 (1.08-1.45)	62	2.42 (1.42-4.11)
Current	2008	2.21 (2.02-2.41)	590	2.86 (2.37-3.45)	580	1.77 (1.50-2.08)	91	4.83 (2.90-8.06)
**Non-Hispanic Black women**								
Never	3186	1 [Reference]	734	1 [Reference]	1014	1 [Reference]	65	1 [Reference]
Former	1320	1.27 (1.17-1.38)	330	1.43 (1.20-1.70)	406	1.20 (1.02-1.40)	63	2.79 (1.70-4.58)
Current	1706	2.20 (2.02-2.41)	532	2.71 (2.31-3.19)	478	2.00 (1.71-2.34)	101	7.73 (5.13-11.63)
**Non-Hispanic White men**								
Never	6766	1 [Reference]	1556	1 [Reference]	2275	1 [Reference]	121	1 [Reference]
Former	11 690	1.32 (1.27-1.37)	3054	1.43 (1.33-1.54)	3681	1.20 (1.13-1.28)	830	4.67 (3.77-5.80)
Current	8110	2.91 (2.79-3.04)	2506	3.68 (3.39-3.98)	2128	2.40 (2.23-2.58)	810	17.92 (14.28-22.48)
**Non-Hispanic White women**								
Never	11 754	1 [Reference]	2583	1 [Reference]	3766	1 [Reference]	331	1 [Reference]
Former	7928	1.51 (1.45-1.56)	2099	1.69 (1.57-1.82)	2150	1.34 (1.25-1.43)	828	5.96 (5.11-6.95)
Current	7328	3.15 (3.03-3.28)	2273	3.57 (3.32-3.85)	1611	2.57 (2.39-2.78)	1109	19.10 (16.45-22.19)
**Other non-Hispanic men** ^b^								
Never	444	1 [Reference]	106	1 [Reference]	123	1 [Reference]	13	1 [Reference]
Former	426	1.20 (1.00-1.45)	111	1.51 (1.08-2.10)	135	1.30 (0.94-1.81)	23	1.45 (0.57-3.71)
Current	401	2.16 (1.80-2.58)	107	2.73 (1.93-3.86)	120	2.32 (1.66-3.25)	30	4.84 (1.91-12.29)
**Other non-Hispanic women** ^b^								
Never	778	1 [Reference]	203	1 [Reference]	225	1 [Reference]	18	1 [Reference]
Former	235	1.68 (1.37-2.06)	53	1.52 (0.99-2.32)	55	1.31 (0.85-2.00)	16	4.19 (1.62-10.84)
Current	247	2.19 (1.80-2.67)	76	2.32 (1.64-3.28)	65	2.17 (1.45-3.23)	28	11.78 (4.12-33.68)
**All men**								
Never	10 224	1 [Reference]	2293	1 [Reference]	3363	1 [Reference]	192	1 [Reference]
Former	15 032	1.30 (1.26-1.34)	3874	1.41 (1.32-1.50)	4824	1.21 (1.15-1.28)	962	3.87 (3.24-4.63)
Current	11 536	2.70 (2.60-2.80)	3474	3.40 (3.18-3.65)	3090	2.27 (2.14-2.42)	975	14.06 (11.64-16.98)
**All women**								
Never	18 125	1 [Reference]	4120	1 [Reference]	5684	1 [Reference]	469	1 [Reference]
Former	10 105	1.46 (1.41-1.50)	2634	1.61 (1.51-1.71)	2774	1.30 (1.22-1.38)	936	5.40 (4.70-6.19)
Current	9848	2.90 (2.80-3.00)	3038	3.29 (3.09-3.50)	2299	2.44 (2.29-2.60)	1269	16.95 (14.75-19.36)
**All participants**								
Never	28 349	1 [Reference]	6413	1 [Reference]	9047	1 [Reference]	661	1 [Reference]
Former	25 137	1.37 (1.34-1.40)	6508	1.52 (1.46-1.59)	7598	1.23 (1.18-1.28)	1898	4.76 (4.26-5.31)
Current	21 384	2.80 (2.73-2.88)	6512	3.38 (3.22-3.55)	5389	2.34 (2.24-2.45)	2244	16.12 (14.48-17.95)

Abbreviation: RR, rate ratio.

^a^ Mortality RRs were adjusted for age at risk (in 5-year groups; ages 25-89 years), educational level, and alcohol consumption.

^b^ Other non-Hispanic race and ethnicity includes any group other than Hispanic, non-Hispanic Black, or non-Hispanic White.

### Sensitivity Analyses

The influence of classifying those who quit smoking in the 5 years preceding death as former smokers (instead of as current smokers) was primarily limited to those quitting at older ages (eFigure 3 in the Supplement). When restricting analyses to participants without major chronic disease at recruitment, the former vs never all-cause mortality RRs were further reduced (eFigure 4 in the Supplement). Associations between never and former or current smoking were similar when separated by follow-up time (<5, 5 to <10, or ≥10 years) (eTable 2 in the Supplement). When further adjusting race- and ethnicity-specific analyses for physical activity, associations were not materially changed (eTable 3 in the Supplement). In addition, analyses among current daily smokers stratified by age of smoking initiation and amount smoked suggest that if smoking habits were similar across racial and ethnic groups, RRs for lower vs higher smoking exposure would be generally similar across these groups (eTable 4 in the Supplement).

### Smoking-Attributable Mortality According to Race, Ethnicity, and Sex

Table 3 shows the estimated race-, ethnicity-, and sex-specific proportion of deaths associated with cigarette smoking among ever smokers and among the general population, including deaths from all causes, cancer, cardiovascular disease, and lower respiratory disease. Among ever smokers overall, an estimated 44.0% of deaths from any cause, 52.2% of deaths from cancer, 34.7% of deaths from cardiovascular disease, and 86.9% of deaths from lower respiratory disease were attributable to smoking. However, the population-attributable fraction in the overall population varied considerably by race, ethnicity, and sex owing to differences in both mortality RRs and ever-smoking prevalence. Overall, an estimated 31.3% of deaths among men and 22.3% of deaths among women were attributable to smoking; race-, ethnicity-, and sex-specific estimates ranged from 10.5% of deaths among Hispanic women to 24.4% of deaths among White women and from 19.8% of deaths among Hispanic men to 33.2% of deaths among White men.

**Table 3.** Adjusted Cause-Specific and All-Cause Mortality RRs, AFs, and PAFs for Ever vs Never Smoking by Race, Ethnicity, and Sex

Cause of death, race and ethnicity	Men and women		Men		Women	
	RR (95% CI)	AF, %^a^	Prevalence, %	PAF, %^b^	Prevalence, %	PAF, %^b^
Cancer						
Hispanic	1.52 (1.33-1.74)	34.2	61.0	20.9	32.3	11.1
Non-Hispanic Black	2.03 (1.83-2.25)	50.7	73.3	37.2	52.5	26.6
Non-Hispanic White	2.17 (2.07-2.27)	53.9	77.1	41.5	62.4	33.6
Other^c^	1.95 (1.57-2.41)	48.7	66.0	32.1	36.1	17.6
Total	2.09 (2.01-2.18)	52.2	75.3	39.3	58.5	30.5
Cardiovascular disease						
Hispanic	1.47 (1.30-1.65)	31.8	60.3	19.2	29.5	9.4
Non-Hispanic Black	1.49 (1.36-1.64)	33.1	65.2	21.5	44.1	14.6
Non-Hispanic White	1.53 (1.47-1.60)	34.8	70.1	24.4	47.2	16.4
Other^c^	1.64 (1.32-2.03)	38.9	65.5	25.5	32.1	12.5
Total	1.53 (1.48-1.59)	34.7	68.8	23.9	45.3	15.7
Lower respiratory disease						
Hispanic	4.09 (2.70-6.19)	75.5	77.8	58.7	51.7	39.1
Non-Hispanic Black	4.07 (3.01-5.50)	75.4	81.5	61.4	70.9	53.5
Non-Hispanic White	8.70 (7.74-9.79)	88.5	92.7	82.1	84.3	74.6
Other^c^	4.04 (2.18-7.51)	75.3	75.8	57.1	64.8	48.8
Total	7.65 (6.91-8.47)	86.9	91.1	79.2	82.2	71.5
All causes						
Hispanic	1.50 (1.40-1.61)	33.4	59.3	19.8	31.4	10.5
Non-Hispanic Black	1.67 (1.59-1.76)	40.2	67.7	27.3	46.1	18.5
Non-Hispanic White	1.83 (1.79-1.87)	45.4	73.1	33.2	53.7	24.4
Other^c^	1.67 (1.50-1.88)	40.3	63.3	25.5	34.5	13.9
Total	1.78 (1.75-1.82)	44.0	71.3	31.3	50.8	22.3

Abbreviations: AF, attributable fraction; PAF, population-attributable fraction; RR, rate ratio.

^a^ Attributable fractions were calculated as the fraction of deaths estimated to be attributable to smoking among ever smokers.

^b^ Population-attributable fractions estimated the population-level relevance of ever smoking to mortality (including never smokers). Sex-specific PAFs were calculated using race- and ethnicity-specific ever vs never smoking RRs (adjusted for sex, age at risk, educational level, and alcohol consumption) and sex-specific ever smoking prevalence among those who died of each cause.

^c^ Other non-Hispanic race and ethnicity includes any group other than Hispanic, non-Hispanic Black, or non-Hispanic White.

## Discussion

In this nationally representative, prospective cohort study in the US, among men and women from diverse racial and ethnic groups, cigarette smoking was associated with substantial excess mortality, but smoking cessation was associated with a substantial reversal of risk. Compared with never smokers, current smokers had 3 times the overall mortality rate among White individuals and approximately twice the mortality rate among Black, Hispanic, and other non-Hispanic individuals, with similar relative excess mortality by sex within each racial and ethnic group after adjusting for age, educational level, and alcohol consumption. However, quitting smoking before age 45 years was associated with reductions of approximately 90% of the excess mortality risk associated with continued smoking, and quitting at ages 45 to 64 years was associated with reductions of approximately 66% of this excess risk irrespective of race and ethnicity (Figure 2).

Given the dose-response relationships between age at quitting smoking and years since quitting and mortality and prior evidence of the hazards of smoking and benefits of quitting, the excess risk of mortality among smokers (vs never smokers) in this study was likely to be attributable to smoking. If one were to assume that the associations in this report are causal, then more than 40% of deaths among ever smokers in this study and more than 60% of deaths among current smokers were attributable to smoking. Approximately 70% of deaths from cancer, 60% of deaths from cardiovascular disease, and more than 90% of deaths from lower respiratory disease among current smokers were attributable to smoking. However, on average, quitting smoking was associated with a reduction of approximately 80% of the excess mortality associated with continued smoking, with the largest health gains among those who quit at younger ages.

The overall hazards of smoking among men and women observed in this study were generally similar to those of prior studies in the US,^2,5^ UK,^14,16^ and Australia.^17,18,19^ However, the current vs never smoker mortality RR was highest among non-Hispanic White smokers (3.00; 95% CI, 2.91-3.10) and somewhat lower among individuals who identified as Black, Hispanic, and other non-Hispanic race and ethnicity, who smoked fewer cigarettes per day on average, were less likely to smoke daily, and began smoking at slightly older ages (as in prior studies^9,10,11^). Nevertheless, mortality rates were substantially higher among individuals who smoked than among those who had never smoked, as reported in other studies of comparably low-intensity smoking.^20,21^ Whether these factors may also explain the somewhat greater relative risk of smoking-related diseases among women than among men, as observed in prior research,^2,22,23^ remains unclear.

Despite variation in the relative hazards of smoking, the relative benefits of quitting were consistent among men and women from diverse racial and ethnic groups, particularly among individuals who quit smoking before age 45 years. For example, former smokers who had quit before age 45 years had all-cause mortality rates approximately 10% to 20% higher (eg, 11%; 95% CI, 8%-15% among non-Hispanic White individuals) than those among never smokers for each group considered compared with approximately 100% to 200% higher mortality rates (eg, 200%; 95% CI, 191%-210% among non-Hispanic White individuals) among those who continued to smoke. The large reductions in excess mortality among those who quit before age 45 years observed in this study were consistent for individuals who identified as Black, Hispanic, White, and other non-Hispanic race and ethnicity. These results provide further support for the broad generalizability of the substantial benefits of smoking cessation across diverse groups notwithstanding variation in smoking habits.

Monitoring the association of smoking with mortality by race, ethnicity, and sex is critical to understanding how the US tobacco epidemic continues to evolve over time and who is most affected by the changes. Despite continued decreases in US smoking prevalence in recent decades,^24^ progress has not been equal across demographic groups. Recent progress in raising the quit ratio among US ever smokers overall has been modest,^25^ and the quit ratio has been consistently lower among Black and Hispanic ever smokers than among non-Hispanic White ever smokers.^26^ Although notable steps have been taken in tobacco control policy enactment and enforcement across the US in recent decades, smoking nevertheless remains the leading preventable cause of death, underscoring the potential for further gains.^8^ Broad and equitable tobacco cessation support for individuals who currently smoke, coupled with tobacco control policies that prevent the next generation from starting to smoke, may avert considerable premature smoking-related mortality in the coming decades if successful.

### Strengths and Limitations

A strength of this study is its size, which allowed for reliable estimates of both the hazards of smoking and benefits of quitting by race, ethnicity, and sex, including the benefits of quitting at different ages and for different durations. A further strength is that this study used data from a nationally representative survey, which should have limited selection bias and enhanced the generalizability of our findings.

This study also has limitations. Because information on smoking habits was collected at a single point in time, the impact of smoking cessation or reinitiation after recruitment could not be assessed. Prior studies^16,20,27^ have suggested that some smokers may quit during follow-up, whereas others (typically few) may begin smoking. Thus, both the true hazards of smoking and the true benefits of quitting may be underestimated in this study. Furthermore, participants were categorized into 4 mutually exclusive racial and ethnic groups, which may have contained considerable heterogeneity. However, even without being exhaustive of all backgrounds, these findings suggest broad generalizability of the substantial hazards of smoking and benefits of quitting for smokers of diverse backgrounds in the general US population. To improve generalizability, the main analyses in this study did not separate participants with and without existing disease at recruitment. However, those with existing disease may be both more likely to quit smoking and at greater risk of death during follow-up, which may have further led to underestimation of the benefits of quitting smoking while healthy (although the benefits of quitting even among individuals with major chronic disease are likely substantial^28,29,30^). These analyses did not incorporate geospatial variables, potentially masking important differences in smoking and smoking cessation habits,^31,32^ tobacco control policies,^33^ and related factors at the state and local level. However, the substantial health benefits of quitting smoking are likely to apply across geographic areas nationwide, although geographic disparities in accessing smoking cessation treatment persist.^34^ In addition, residual confounding may have been present even after adjustment for several confounding factors.

## Conclusions

In this prospective cohort study, smoking was associated with substantially greater mortality among female and male current smokers than among never smokers in all racial and ethnic groups considered, but quitting smoking was associated with substantially reversed risks for all groups. Quitting smoking before age 45 years was associated with reductions of approximately 90% of the excess mortality associated with continued smoking, and quitting at ages 45 to 64 years was associated with reductions of approximately 66% of this excess risk.

## Notes

Supplement. **eFigure 1.** All-Cause Mortality Rate Ratios by Years Since Quitting Smoking and by Sex, Compared With Never and Current Smokers, Ages 25-89 **eFigure 2.** All-Cause Mortality Rate Ratios by Years Since Quitting Smoking and Race and Ethnicity, Compared With Never and Current Smokers, Ages 25-89 **eFigure 3.** All-Cause Mortality Rate Ratios by Age at Quitting Smoking and by Sex, Compared With Never and Current Smokers, Ages 25-89 **eFigure 4.** All-Cause Mortality Rate Ratios by Age at Quitting Smoking and by Sex Among Those Without Major Chronic Disease at Recruitment, Compared With Never and Current Smokers, Ages 25-89 **eTable 1.** National Health Interview Survey Questions Used to Determine Smoking Exposure Categories and Relevant Descriptive Statistics **eTable 2.** All-Cause Mortality RRs by Follow-up Period and Smoking Category, Ages 25-89 **eTable 3.** Physical Activity–Adjusted All-Cause Mortality Rate Ratios by Age at Quitting Smoking and Race and Ethnicity, Compared With Never and Current Smokers, Ages 25-89 **eTable 4.** All-Cause Mortality Rate Ratios for Age Began Smoking and Cigarettes per Day Among Current Daily Smokers, by Race and Ethnicity
