# Supplementary material for: Association Between Smoking, Smoking Cessation, and Mortality by Race, Ethnicity, and Sex Among US Adults
Source: JAMA Netw Open. 2022 Oct 24;5(10):e2231480. doi: 10.1001/jamanetworkopen.2022.31480 (PMC9593233; doi:10.1001/jamanetworkopen.2022.31480)
Supplement: Supplement. — eFigure 1. All-Cause Mortality Rate Ratios by Years Since Quitting Smoking and by Sex, Compared With Never and Current Smokers, Ages 25-89 eFigure 2. All-Cause Mortality Rate Ratios by Years Since Quitting Smoking and Race and Ethnicity, Compared With Never and Current Smokers, Ages 25-89 eFigure 3. All-Cause Mortality Rate Ratios by Age at Quitting Smoking and by Sex, Compared With Never and Current Smokers, Ages 25-89 eFigure 4. All-Cause Mortality Rate Ratios by Age at Quitting Smoking and by Sex Among Those Without Major Chronic Disease at Recruitment, Compared With Never and Current Smokers, Ages 25-89 eTable 1. National Health Interview Survey Questions Used to Determine Smoking Exposure Categories and Relevant Descriptive Statistics eTable 2. All-Cause Mortality RRs by Follow-up Period and Smoking Category, Ages 25-89 eTable 3. Physical Activity–Adjusted All-Cause Mortality Rate Ratios by Age at Quitting Smoking and Race and Ethnicity, Compared With Never and Current Smokers, Ages 25-89 eTable 4. All-Cause Mortality Rate Ratios for Age Began Smoking and Cigarettes per Day Among Current Daily Smokers, by Race and Ethnicity [file jamanetwopen-e2231480-s001.pdf]

Supplementary Online Content

Thomson B, Emberson J, Lacey B, et al. Association between smoking, smoking cessation, and mortality by race, ethnicity, and sex among US adults. *JAMA Netw Open*. 2022;5(10):e2231480. doi:10.1001/jamanetworkopen.2022.31480

eFigure 1. All-Cause Mortality Rate Ratios by Years Since Quitting Smoking and by Sex, Compared with Never and Current Smokers, Ages 25-89

eFigure 2. All-Cause Mortality Rate Ratios by Years Since Quitting Smoking and Race and Ethnicity, Compared With Never and Current Smokers, Ages 25-89

eFigure 3. All-Cause Mortality Rate Ratios by Age at Quitting Smoking and by Sex, Compared With Never and Current Smokers, Ages 25-89

eFigure 4. All-Cause Mortality Rate Ratios by Age at Quitting Smoking and by Sex Among Those Without Major Chronic Disease at Recruitment, Compared With Never and Current Smokers, Ages 25-89

eTable 1. National Health Interview Survey Questions Used to Determine Smoking Exposure Categories and Relevant Descriptive Statistics

eTable 2. All-Cause Mortality RRs by Follow-up Period and Smoking Category, Ages 25-89

eTable 3. Physical Activity–Adjusted All-Cause Mortality Rate Ratios by Age at Quitting Smoking and Race and Ethnicity, Compared With Never and Current Smokers, Ages 25-89

eTable 4. All-Cause Mortality Rate Ratios for Age Began Smoking and Cigarettes per Day Among Current Daily Smokers, by Race and Ethnicity

This supplementary material has been provided by the authors to give readers additional information about their work.

© 2022 Thomson B et al. *JAMA Network Open*.

eFigure 1. All-cause mortality rate ratios by years since quitting smoking and by sex, compared with never and current smokers, ages 25−89

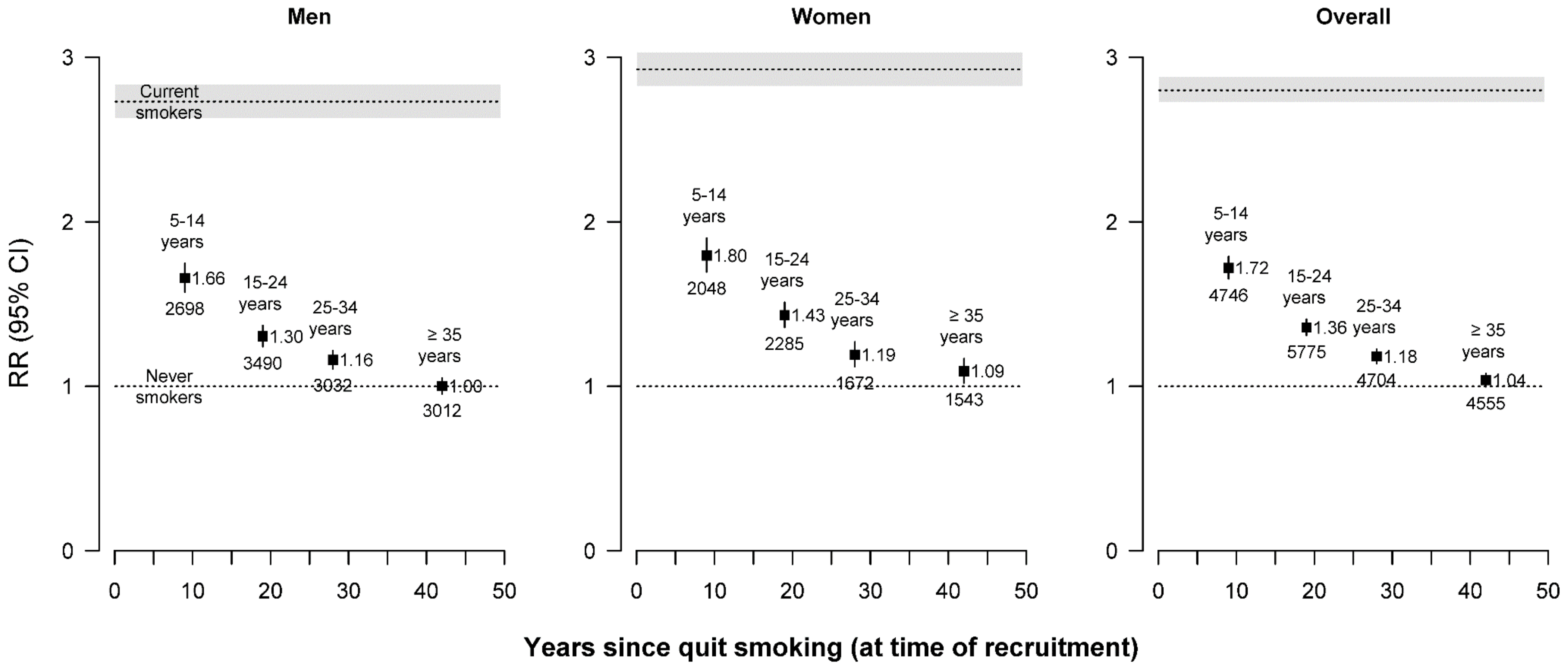

Dotted lines indicate RR for current (top; shaded area 95% CI) and never (bottom) smokers at recruitment.
Number of deaths in each category shown below 95% CI. Adjusted for age-at-risk, sex, education, alcohol consumption, and race.
Note: 1928 deaths among participants who quit smoking <5 years before recruitment but did not die within 5 years of quitting (whether survived or censored) not plotted.

© 2022 Thomson B et al. *JAMA Network Open*.

eFigure 2. All-cause mortality rate ratios by years since quitting smoking and race and ethnicity, compared with never and current smokers, ages 25-89

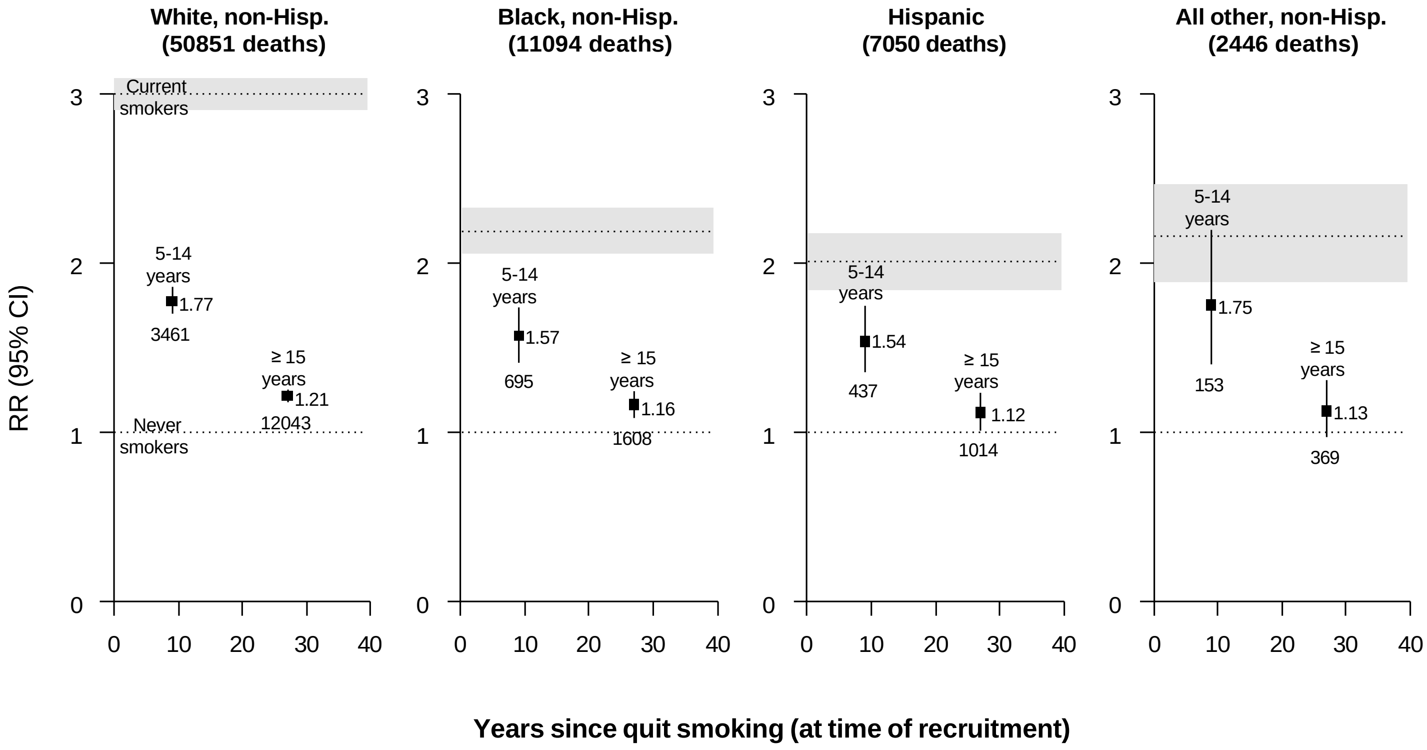

Shaded areas indicate RR (and 95% CI) for current (top) and never (bottom) smokers at recruitment.
Number of deaths in each category shown below 95% CI. Adjusted for age-at-risk, sex, education, and alcohol consumption.
Note: 1928 deaths among participants who quit smoking <5 years before recruitment but did not die within 5 years of quitting (whether survived or censored) not plotted.

© 2022 Thomson B et al. *JAMA Network Open*.

eFigure 3. All-cause mortality rate ratios by age at quitting smoking and by sex, compared with never and current smokers, ages 25−89 (Note: smokers who quit within 5 years of dying not re−categorized as current smokers)

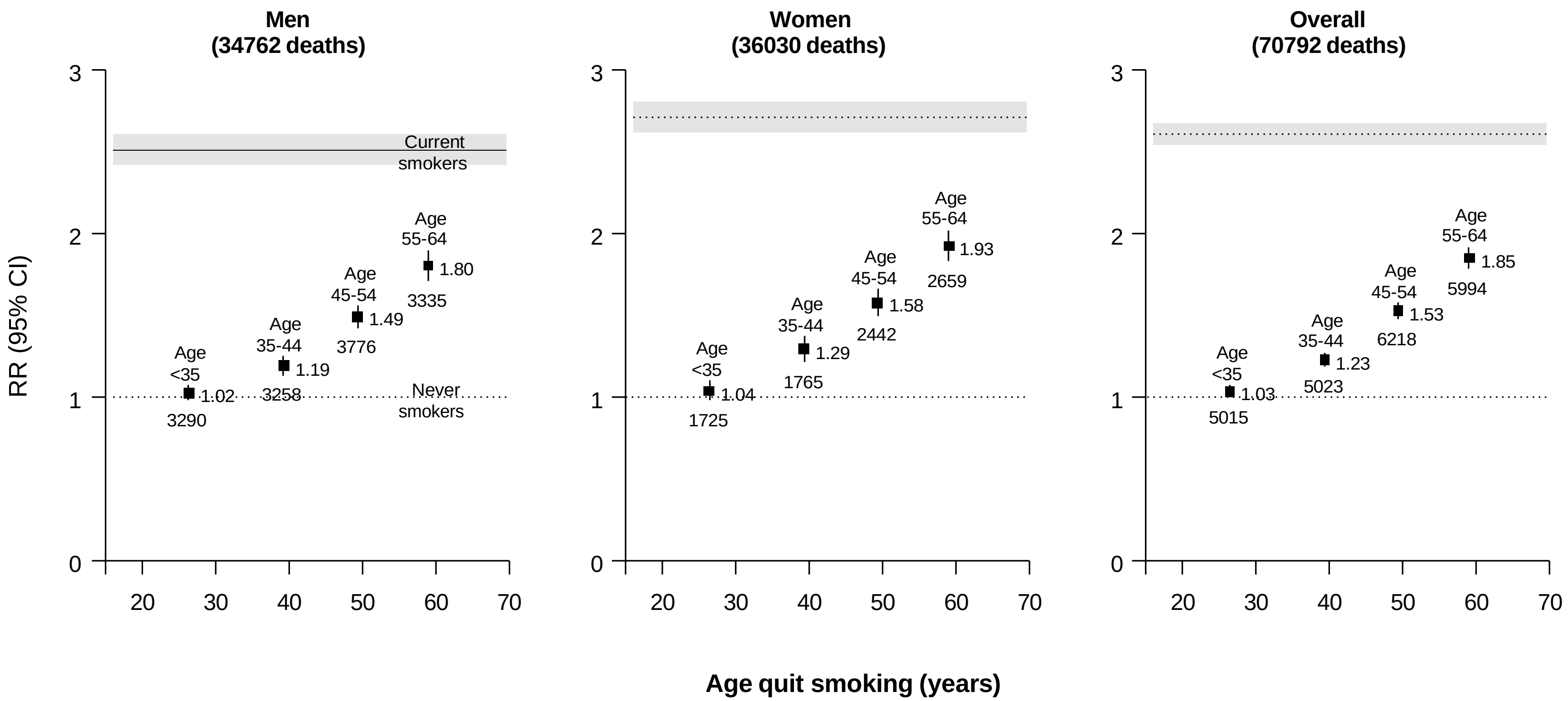

Shaded areas indicate RR (and 95% CI) for current (top) and never (bottom) smokers at recruitment.
Number of deaths in each category shown below 95% CI. Adjusted for age-at-risk, sex, education, alcohol consumption, and race.
Note: The 515 fewer deaths than in Figure 1 occurred among those who quit aged>65 years and died within 5 years of quitting.

© 2022 Thomson B et al. *JAMA Network Open*.

eFigure 4. All-cause mortality rate ratios by age at quitting smoking and by sex among those without major chronic disease at recruitment, compared with never and current smokers, ages 25-89

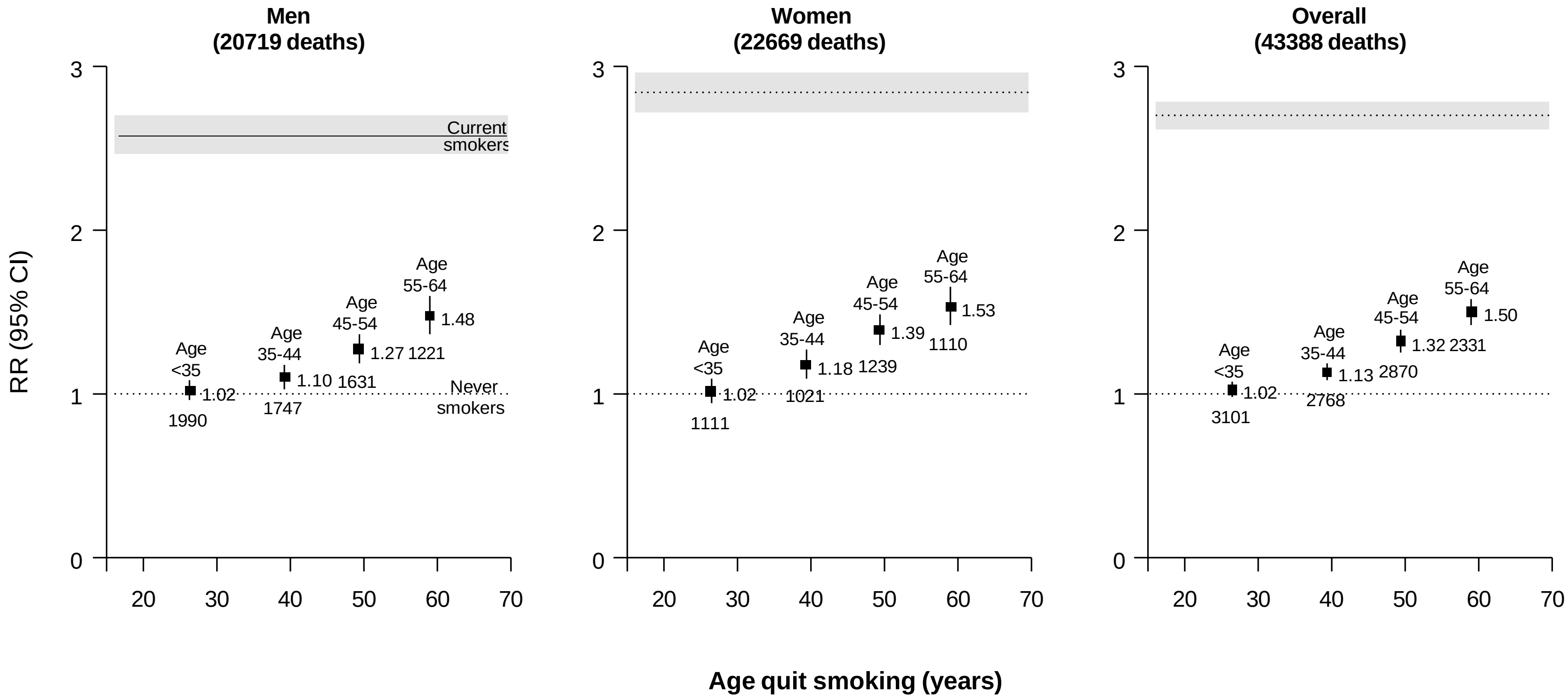

Shaded areas indicate RR (and 95% CI) for current (top) and never (bottom) smokers at recruitment.
Number of deaths in each category shown below 95% CI. Adjusted for age-at-risk, sex, education, alcohol consumption, and race.
Major chronic disease was self-reported, and included heart disease, stroke, cancer, and emphysema.

© 2022 Thomson B et al. *JAMA Network Open*.

eTable 1. National Health Interview Survey questions used to determine smoking exposure categories and relevant descriptive statistics

Survey years	Smoking category	Question text
1997-2018	Never/ever smoker	Have you smoked at least 100 cigarettes in your ENTIRE LIFE?
1997-2018	Ever smoker	How old were you when you FIRST started to smoke fairly regularly?
1997-2018	Current (every day or some days) or ex-smoker	Do you NOW smoke cigarettes every day, some days, or not at all?
1997-2018	Ex-smoker	How long has it been since you quit smoking cigarettes?
1997-2018	Current (every day or some days) smoker	On the average, how many cigarettes do you now smoke a day?
1997-2018	Current (some days) smoker	On how many of the PAST 30 DAYS did you smoke a cigarette?
1997-2018	Current (some days) smoker	On the average, when you smoked during the PAST 30 DAYS, about how many cigarettes did you smoke a day?

For the main analyses, former smokers who quit within 5 years of death were re-classified as current smokers. For example, individuals who quit smoking 1 year before recruitment and died after 2 years of follow-up were considered current smokers in these analyses (to limit reverse causality, ie, poor health causing smokers to quit).

© 2022 Thomson B et al. *JAMA Network Open*.

eTable 2. All-cause mortality RRs by follow-up period and smoking category, ages 25-89

**Follow-up period**	**Years 0-<5 of follow-up**	**Years 5-<10 of follow-up**	**Years ≥10 of follow-up**
	**RR (95% CI)**	**RR (95% CI)**	**RR (95% CI)**
Never smoker	1 (ref)	1 (ref)	1 (ref)
Quit age <35 years	1.02 (0.98-1.04)	1.03 (0.99-1.07)	1.05 (0.99-1.11)
Quit age 35-44	1.22 (1.18-1.26)	1.24 (1.19-1.29)	1.21 (1.14-1.28)
Quit age 45-54	1.41 (1.37-1.46)	1.42 (1.37-1.47)	1.46 (1.38-1.55)
Quit age 55-64	1.53 (1.51-1.60)	1.62 (1.55-1.68)	1.76 (1.65-1.88)
Current smoker	2.57 (2.51-2.63)	2.48 (2.41-2.55)	2.55 (2.45-2.66)

Adjustments made as in the main analyses. Due to distinct model fitting and weighting, RRs are slightly different than when modeled jointly, as in main analyses.

© 2022 Thomson B et al. *JAMA Network Open*.

eTable 3. Physical activity–adjusted all-cause mortality rate ratios by age at quitting smoking and race and ethnicity, compared with never and current smokers, ages 25-89

Smoking category	Non-Hisp. White (RR 95% CI)	Non-Hisp. Black (RR 95% CI)	Hispanic (RR 95% CI)	All other non-Hisp. (RR 95% CI)
Never smoker	1.00 (ref)	1.00 (ref)	1.00 (ref)	1.00 (ref)
Quit age <45 years	1.13 (1.10-1.17)	1.17 (1.08-1.26)	1.16 (1.04-1.30)	1.21 (1.02-1.43)
Quit age 45-64 years	1.63 (1.58-1.68)	1.41 (1.31-1.52)	1.31 (1.18-1.45)	1.42 (1.19-1.70)
Current smoker	2.85 (2.77-2.94)	2.15 (2.02-2.29)	1.96 (1.80-2.13)	2.08 (1.81-2.38)

Adjustments made as in the main analyses, further adjusting for five categories of leisure time physical activity per week: missing, 0 minutes, >0 to <150 minutes, 150-300 minutes, or >300 minutes. One minute of vigorous activity counted as equivalent to two minutes of moderate activity, as per 2008 guidelines (https://health.gov/sites/default/files/2019-09/paguide.pdf). Code and further description provided at https://www.cdc.gov/nchs/nhis/physical_activity/pa_recodes.htm.

© 2022 Thomson B et al. *JAMA Network Open*.

eTable 4. All-cause mortality rate ratios for age began smoking and cigarettes per day among current daily smokers, by race and ethnicity

Smoking category	Non-Hisp. White (RR 95% CI)	Non-Hisp. Black (RR 95% CI)	Hispanic (RR 95% CI)	All other non-Hisp. (RR 95% CI)
Began age ≥15 years, <20 cigs/day	1.00 (ref)	1.00 (ref)	1.00 (ref)	1.00 (ref)
Began age <15 years, <20 cigs/day	1.33 (1.21-1.48)	1.20 (1.01-1.44)	1.09 (0.87-1.37)	1.04 (0.68-1.59)
Began age ≥15 years, ≥20 cigs/day	1.26 (1.19-1.32)	1.27 (1.13-1.44)	1.39 (1.15-1.68)	1.10 (0.84-1.44)
Began age <15 years, ≥20 cigs/day	1.52 (1.42-1.62)	1.50 (1.25-1.81)	1.65 (1.29-2.11)	1.20 (0.85-1.71)

Above analyses also adjusted for age-at-risk, sex, education, and alcohol consumption.

© 2022 Thomson B et al. *JAMA Network Open*.
